# Relationship between the COVID-19 pandemic and structural inequalities within the pediatric trauma population

**DOI:** 10.1186/s40621-023-00475-0

**Published:** 2023-11-28

**Authors:** Christina Georgeades, Amelia T. Collings, Manzur Farazi, Carisa Bergner, Mary E. Fallat, Peter C. Minneci, K. Elizabeth Speck, Kyle J. Van Arendonk, Katherine J. Deans, Richard A. Falcone, David S. Foley, Jason D. Fraser, Samir K. Gadepalli, Martin S. Keller, Meera Kotagal, Matthew P. Landman, Charles M. Leys, Troy A. Markel, Nathan S. Rubalcava, Shawn D. St. Peter, Thomas T. Sato, Katherine T. Flynn-O’Brien, the Midwest Pediatric Surgery Consortium (MWPSC)

**Affiliations:** 1 Department of Surgery, Medical College of Wisconsin, Milwaukee, WI USA; 2 Division of Pediatric Surgery, Medical College of Wisconsin, Children’s Corporate Center, Suite C320, 999 N 92Nd St, Milwaukee, WI 53226 USA; 3 Department of Surgery, Indiana University, Indianapolis, IN USA; 4 Norton Children’s Hospital, Louisville, KY USA; 5 Hiram C. Polk Jr., Department of Surgery, University of Louisville, Louisville, KY USA; 6 Center for Surgical Outcomes Research, Abigail Wexner Research Institute and Department of Surgery, Nationwide Children’s Hospital, The Ohio State University College of Medicine, Columbus, OH USA; 7 Division of Pediatric Surgery, Mott Children’s Hospital, Ann Arbor, MI USA; 8 Department of Surgery, Nemours Children’s Health Delaware Valley, Wilmington, DE USA; 9 Division of Pediatric General and Thoracic Surgery, Cincinnati Children’s Hospital Medical Center, Cincinnati, OH USA; 10 Department of Surgery, University of Cincinnati College of Medicine, Cincinnati, OH USA; 11 Department of Surgery, Children’s Mercy Hospital, Kansas City, MO USA; 12 Division of Pediatric Surgery, Washington University School of Medicine, St Louis, MO USA; 13 Division of Pediatric Surgery, Department of Surgery, University of Wisconsin, Madison, WI USA

**Keywords:** Pediatric trauma, Violent injury, Socioeconomic status, Race/ethnicity

## Abstract

**Background:**

The COVID-19 pandemic disrupted social, political, and economic life across the world, shining a light on the vulnerability of many communities. The objective of this study was to assess injury patterns before and after implementation of stay-at-home orders (SHOs) between White children and children of color and across varying levels of vulnerability based upon children’s home residence.

**Methods:**

A multi-institutional retrospective study was conducted evaluating patients < 18 years with traumatic injuries. A “Control” cohort from an averaged March-September 2016–2019 time period was compared to patients injured after SHO initiation-September 2020 (“COVID” cohort). Interactions between race/ethnicity or social vulnerability index (SVI), a marker of neighborhood vulnerability and socioeconomic status, and the COVID-19 timeframe with regard to the outcomes of interest were assessed using likelihood ratio Chi-square tests. Differences in injury intent, type, and mechanism were then stratified and explored by race/ethnicity and SVI separately.

**Results:**

A total of 47,385 patients met study inclusion. Significant interactions existed between race/ethnicity and the COVID-19 SHO period for intent (*p* < 0.001) and mechanism of injury (*p* < 0.001). There was also significant interaction between SVI and the COVID-19 SHO period for mechanism of injury (*p* = 0.01). Children of color experienced a significant increase in intentional (COVID 16.4% vs. Control 13.7%, *p* = 0.03) and firearm (COVID 9.0% vs. Control 5.2%, *p* < 0.001) injuries, but no change was seen among White children. Children from the most vulnerable neighborhoods suffered an increase in firearm injuries (COVID 11.1% vs. Control 6.1%, *p* = 0.001) with children from the least vulnerable neighborhoods having no change. All-terrain vehicle (ATV) and bicycle crashes increased for children of color (COVID 2.0% vs. Control 1.1%, *p* = 0.04 for ATV; COVID 6.7% vs. Control 4.8%, *p* = 0.02 for bicycle) and White children (COVID 9.6% vs. Control 6.2%, *p* < 0.001 for ATV; COVID 8.8% vs. Control 5.8%, *p* < 0.001 for bicycle).

**Conclusions:**

In contrast to White children and children from neighborhoods of lower vulnerability, children of color and children living in higher vulnerability neighborhoods experienced an increase in intentional and firearm-related injuries during the COVID-19 pandemic. Understanding inequities in trauma burden during times of stress is critical to directing resources and targeting intervention strategies.

**Supplementary Information:**

The online version contains supplementary material available at 10.1186/s40621-023-00475-0.

## Introduction

The COVID-19 pandemic disrupted social, political, and economic life across the world, highlighting the vulnerability of many communities and the children within them. Changes in daily life occurred with the implementation of stay-at-home orders (SHOs), as children no longer had access to school or extracurricular activities. Many experienced social isolation in addition to an increase in family-related stress due to parental unemployment and financial strain (Pereda and Díaz-Faes 2020; Nearchou et al. 2020; Smith et al. 2020). The COVID-19 pandemic further exacerbated difficulties experienced by certain racial/ethnic groups and those living in vulnerable neighborhoods (Fraiman et al. 2021; Gati et al. 2020; Abrams et al. 2022). Prior to the pandemic, social determinants of health, neighborhood-level factors, and socioeconomic hardship were known to influence trends in pediatric trauma for vulnerable populations (Marcin et al. 2003; Trinidad et al. 2022a, 2022b). These contributing factors have persisted throughout the COVID-19 pandemic.

Few studies have evaluated the impact of COVID-19 and the initiation of SHOs on pediatric traumatic injuries specifically related to race/ethnicity and neighborhood-level characteristics, a surrogate marker for socioeconomic status (SES). Existing literature is narrow in scope, mostly focusing on firearm injuries and having mixed results (Afif et al. 2022; Collings et al. 2022; Martin et al. 2022; Gastineau et al. 2021; Peña and Jena 2022). Other studies have assessed race/ethnicity and vulnerable population differences for the entire trauma cohort rather than by individual intents, types, and mechanisms of injury (Flynn-O’Brien et al. 2022a; Bessoff et al. 2021; Sanford et al. 2021; Yeates et al. 2022). Our previous work suggested that a disparate burden of violent injuries was sustained by children of color and children living in higher vulnerability neighborhoods during the first six months of the COVID-19 pandemic (Collings et al. 2022; Flynn-O’Brien et al. 2022b).

The objective of this study was to assess injury patterns before and after implementation of SHOs during the COVID-19 pandemic by race/ethnicity and by social vulnerability. We hypothesized that the relationship between the COVID-19 pandemic and intent, type, and mechanism of injury would be different for children of color and children living in areas of higher vulnerability. Specifically, we hypothesized that children of color and children living in areas of higher vulnerability experienced an increase in primarily violent injuries, while White children and children living in areas of lower vulnerability experienced instead an increase in primarily non-violent injuries.

## Methods

### Study design and population

A secondary analysis of a retrospective multi-institutional cohort study was completed to compare the differences in injury patterns before and after implementation of SHOs for White children and children of color, and for children living in neighborhoods of varying vulnerability as measured by the social vulnerability index (SVI).

Children < 18 years of age with traumatic injuries at nine Level 1 Pediatric Trauma Centers were included. All patients met National Trauma Data Bank (NTDB) inclusion criteria. Patients that had at least one traumatic injury were identified with the following International Classification of Diseases, 10th revision (ICD-10), diagnosis codes: ICD-10-CM S00-S99, T07, T14, T20-T28, T30-T32, and T79.A1-T79.A9. Repeat visits to the emergency department were not included per NTDB inclusion and exclusion guidelines (American College of Surgeons 2022). For the purpose of this study, the start of the COVID-19 pandemic was defined as the initiation of SHOs. Children injured after implementation of SHOs at each center, which included varying dates (concordant with each regional SHOs at each site) in March and early April 2020, through the end of September 2020 defined the ‘COVID cohort.’ Historical controls from March to September over four consecutive years (2016–2019) were the ‘Control cohort.’ Injury rates within this cohort were averaged to minimize outlying effects from any one year. This study was approved by the Institutional Review Board at each center with a waiver of consent.

### Data definitions

#### Race/ethnicity and Social Vulnerability Index

‘Children of color’ was defined by self-reported race and/or ethnicity and included patients who identified as Black, Hispanic/Latino, Asian, Hawaiian/Pacific Islander, Native American, or ‘other.’ The term ‘children of color’ was used as opposed to the term ‘minority’ or ‘non-White’ since the latter terms are reflective of deficit language in relation to White children (Flanagin et al. 2021; National Museum of African American History & Culture 2023; Utah Department of Cultural Community Engagement 2019). Children in the ‘White’ classification self-reported as non-Hispanic White. Within the study population, 6.3% (815/12,959 children) had unknown race and/or ethnicity, such that they could not be appropriately classified and were excluded from the race/ethnicity-based analysis.

Social vulnerability was examined to determine how the COVID-19 pandemic impacted traumatically injured children based on social determinants of health. The Centers for Disease Control and Prevention’s SVI measures a community’s ability to function during a disaster. Each census tract is ranked on 15 social factors that encompass four domains: SES, household composition and disability, racial and ethnic minority status and language, and housing and transportation (Agency for Toxic Substances and Disease Registry 2022). These factors are obtained from 2018 American Community Survey data, which is released by the United States Census Bureau every two years. An SVI of 0 represents the population with the lowest vulnerability and an SVI of 1 represents the highest vulnerability. SVI was separated into four quartiles (0–0.249, 0.250–0.499, 0.500–0.749, 0.750–1.00), with the first quartile representing the least vulnerable children and the fourth quartile representing the most vulnerable children.

#### Injury characteristics

Injury characteristics included intent, type, and mechanism of injury. Intent of injury included unintentional injuries, intentional injuries, and unknown intents. Intentional injuries included assault, suicide, and other intentional injuries. Type of injury included blunt, burn, penetrating, and unknown injury types. Mechanism of injury included child abuse, falls, firearm injuries, and motor vehicle crashes (MVCs) among others. Injury severity score (ISS) was divided into mild (0–14), moderate (15–24), and severe (≥ 25). Mortality included death in the emergency department or during the hospital stay. For the purpose of this study, ‘violent’ injuries included intentional and firearm injuries. Other types and mechanisms of injury such as penetrating and cut/pierce injuries, although potentially violent in nature, may not necessarily be so. Unintentional injuries and MVCs (among other mechanisms) were considered to be non-violent.

### Statistical analysis

To determine whether the association between the COVID-19 pandemic SHOs and the intent, type, and mechanism of injury varied based upon race/ethnicity and social vulnerability, likelihood ratio Chi-squares were used to determine whether there was statistical interaction (i.e., effect modification). Given the significance of interactive effects for race/ethnicity and social vulnerability, stratified analyses were then conducted to investigate the relationship between SHOs and intent, type, and mechanism of injury separately for White children and children of color and separately for children living in varying degrees of social vulnerability.

For the stratified analysis, Chi-squared tests were used to evaluate categorical variables between cohorts, stratified by race/ethnicity or SVI. Fisher’s exact tests were used for cell sizes < 5. When a variable with more than two groups was statistically significant, comparisons of binomial proportions were completed to understand which category was statistically significant. Statistical significance was set at *p* < 0.05. Missing data were presented only if accounting for > 5% of categories.

To further explore the hypothesis that children of color disproportionately suffered from violent injuries, an interrupted time series analysis (ITSA) was performed to specifically evaluate the variation in expected and observed rates of intentional injuries per month in children of color and White children. Because SHO dates were not simultaneous across sites, the ‘interruption point’ used was March 13, 2020 as this was the declaration of a state of emergency in the USA regarding the COVID-19 pandemic. The dates of SHO implementation for each site in the study occurred after the state of emergency was declared. Kolmogorov–Smirnov testing was used to evaluated differences in density distribution (Pratt and Gibbons 1981). Statistical analysis was performed using RStudio©, version 1.4.1717.

## Results

### Overall study population and tests of interaction

A total of 47,385 pediatric trauma patients met study inclusion. There were 7,068 patients within the COVID cohort and an average of 5,891 patients in the Control cohort, showing an overall significant increase in pediatric trauma volume (*p* = 0.03). The likelihood ratio Chi-square tests established significant interaction between the COVID-19 SHO period and race/ethnicity on intent (*p* < 0.001) and mechanism of injury (*p* < 0.001). There was also a significant interaction between SVI and the COVID-19 SHO period on mechanism of injury (*p* = 0.01). Due to significance of interactive effects (Additional file 1), stratified analyses were completed. Comparisons between the COVID and Control cohorts by race/ethnicity are displayed in Table 1 and by SVI quartiles in Table 2.

**Table 1:** Injury characteristics by race/ethnicity

	Children of Color^a,b^			White Children^b^		
	Control *N* = 1580	COVID *N* = 1888	*p* value	Control *N* = 3703	COVID *N* = 4973	*p* value
Intent of Injury, *N* (%)						
Intentional	216 (13.7)	310 (16.4)	**0.03**	192 (5.2)	217 (4.4)	0.08
Unintentional	1344 (85.1)	1576 (83.5)	0.22	3471 (93.7)	4750 (95.5)	** < 0.001**
Unknown	20 (1.3)	2 (0.1)	** < 0.001**	40 (1.1)	6 (0.1)	** < 0.001**
Type of Injury, *N* (%)						
Blunt	1179 (74.7)	1342 (71.1)	**0.02**	3051 (82.4)	4068 (81.8)	0.48
Burn	120 (7.6)	156 (8.3)	0.51	172 (4.7)	307 (6.2)	**0.002**
Penetrating	166 (10.5)	305 (16.2)	** < 0.001**	259 (7.0)	442 (8.9)	**0.002**
Unknown	114 (7.2)	85 (4.5)	**0.001**	220 (5.9)	156 (3.2)	** < 0.001**
Mechanism of Injury, *N* (%)						
Burn	120 (7.6)	156 (8.3)	0.52	172 (4.6)	307 (6.2)	**0.002**
Child Abuse	88 (5.6)	109 (5.8)	0.86	118 (3.2)	140 (2.8)	0.35
Cut/Pierce	52 (3.3)	78 (4.1)	0.23	108 (2.9)	160 (3.2)	0.46
Fall	570 (36.1)	607 (32.2)	**0.02**	1652 (44.6)	1969 (39.6)	** < 0.001**
Firearm	82 (5.2)	170 (9.0)	** < 0.001**	19 (0.5)	33 (0.7)	0.45
Motor Vehicle Crash	210 (13.3)	264 (14.0)	0.60	399 (10.8)	407 (8.2)	** < 0.001**
All-Terrain Vehicle	17 (1.1)	38 (2.0)	**0.04**	229 (6.2)	471 (9.6)	** < 0.001**
Bicycle	76 (4.8)	126 (6.7)	**0.02**	215 (5.8)	439 (8.8)	** < 0.001**
Pedestrian	80 (5.1)	54 (2.9)	**0.001**	69 (1.9)	76 (1.5)	0.26
Struck By/Against	142 (9.0)	107 (5.7)	** < 0.001**	334 (9.0)	377 (7.6)	**0.02**
Unknown	141 (8.9)	179 (9.5)	0.62	388 (10.5)	594 (11.9)	**0.04**
Injury Severity Score, *N* (%)						
0–14	1375 (91.8)	1691 (90.4)	0.35	3209 (92.6)	4611 (93.0)	0.74
15–24	74 (4.9)	105 (5.6)		164 (4.7)	225 (4.5)	
≥ 25	49 (3.3)	75 (4.0)		93 (2.7)	122 (2.5)	
Mortality, *N* (%)						
Alive	1556 (98.5)	1851 (98.0)	0.39	3678 (99.3)	4949 (99.5)	0.30
Dead	24 (1.5)	37 (2.0)		25 (0.7)	24 (0.5)	

Bold* p*-values represent statistically significant results

^a^Children of color = children that self-reported as Black, Hispanic/Latino, Asian, Hawaiian/Pacific Islander, Native American, or ‘other’

^b^When a variable with more than two groups was statistically significant, comparisons of binomial proportions were completed to understand which category was statistically significant

**Table 2:** Injury characteristics by social vulnerability index^a^

	First Quartile^b^			Second Quartile^b^			Third Quartile^b^			Fourth Quartile^b^		
	Control *N* = 1211	COVID *N* = 1590	*p* value	Control *N* = 2034	COVID *N* = 2426	*p* value	Control *N* = 1953	COVID *N* = 2232	*p* value	Control *N* = 693	COVID *N* = 820	*p* value
Intent of Injury, *N* (%)												
Intentional	58 (4.8)	62 (3.9)	0.29	129 (6.3)	154 (6.4)	1.00	160 (8.2)	189 (8.5)	0.80	102 (14.7)	147 (17.9)	0.11
Unintentional	1139 (94.1)	1526 (96.0)	**0.02**	1877 (92.3)	2269 (93.5)	0.12	1774 (90.9)	2040 (91.4)	0.59	583 (84.1)	673 (82.1)	0.32
Unknown	14 (1.2)	2 (0.1)	**0.001**	28 (1.4)	3 (0.1)	**< 0.001**	18 (0.9)	3 (0.1)	**0.001**	8 (1.2)	0 (0.0)	**0.006**
Type of Injury, *N* (%)												
Blunt	1031 (85.1)	1347 (84.7)	0.10	1653 (81.3)	1932 (79.6)	0.17	1537 (78.7)	1720 (77.1)	0.21	509 (73.3)	569 (69.4)	0.10
Burn	48 (4.0)	79 (5.0)		102 (5.0)	156 (6.4)	0.05	115 (5.9)	174 (7.8)	**0.02**	52 (7.5)	66 (8.1)	0.76
Penetrating	70 (5.8)	107 (6.7)		150 (7.4)	263 (10.8)	**< 0.001**	172 (8.8)	253 (11.3)	**0.008**	83 (12.0)	153 (18.7)	**< 0.001**
Unknown	62 (5.1)	57 (3.6)		128 (6.3)	75 (3.1)	**< 0.001**	128 (6.6)	85 (3.8)	**< 0.001**	50 (7.2)	32 (3.9)	**0.007**
Mechanism of Injury, *N* (%)												
Burn	48 (4.0)	79 (5.0)	0.24	102 (5.0)	156 (6.4)	0.05	114 (5.8)	174 (7.8)	**0.02**	52 (7.5)	66 (8.1)	0.77
Child Abuse	30 (2.5)	28 (1.8)	0.24	67 (3.3)	78 (3.2)	0.95	90 (4.61)	107 (4.8)	0.83	42 (6.1)	51 (6.2)	0.98
Cut/Pierce	32 (2.6)	43 (2.7)	1.00	63 (3.1)	101 (4.2)	0.07	64 (3.3)	77 (3.5)	0.82	24 (3.5)	30 (3.7)	0.95
Fall	604 (49.9)	737 (46.4)	0.07	866 (42.6)	913 (37.6)	**0.001**	796 (40.8)	790 (35.4)	**< 0.001**	217 (31.3)	212 (25.9)	**0.02**
Firearm	8 (0.7)	7 (0.4)	0.60	19 (0.9)	48 (2.0)	**0.006**	38 (2.0)	69 (3.1)	**0.03**	42 (6.1)	91 (11.1)	**0.001**
Motor Vehicle Crash	113 (9.3)	105 (6.6)	**0.009**	231 (11.4)	222 (9.2)	**0.02**	218 (11.2)	224 (10.0)	0.26	109 (15.7)	138 (16.8)	0.61
All-Terrain Vehicle	52 (4.3)	104 (6.5)	**0.01**	109 (5.4)	224 (9.2)	**< 0.001**	106 (5.4)	170 (7.6)	**0.005**	14 (2.0)	27 (3.3)	0.17
Bicycle	74 (6.1)	177 (11.1)	**< 0.001**	114 (5.6)	205 (8.5)	**< 0.001**	104 (5.3)	139 (6.2)	0.24	32 (4.6)	53 (6.5)	0.15
Pedestrian	18 (1.5)	18 (1.1)	0.51	40 (2.0)	36 (1.5)	0.26	60 (3.1)	46 (2.1)	0.05	43 (6.2)	35 (4.3)	0.11
Struck By/Against	116 (9.6)	124 (7.8)	0.11	205 (10.1)	164 (6.8)	**< 0.001**	154 (7.9)	164 (7.4)	0.55	58 (8.4)	42 (5.1)	**0.02**
Unknown	116 (9.6)	168 (10.6)	0.43	217 (10.7)	279 (11.5)	0.41	209 (10.7)	272 (12.2)	0.15	60 (8.7)	75 (9.2)	0.81
Injury Severity Score, *N* (%)												
0–14	1054 (93.1)	1491 (94.1)	0.34	1732 (92.5)	2238 (92.5)	0.99	1734 (92.4)	2026 (91.2)	0.15	612 (91.3)	735 (90.6)	0.78
15–24	48 (4.2)	64 (4.0)		88 (4.7)	112 (4.6)		94 (5.0)	116 (5.2)		31 (4.6)	44 (5.4)	
≥ 25	30 (2.7)	29 (1,8)		53 (2.8)	69 (2.9)		48 (2.6)	80 (3.6)		27 (4.0)	32 (4.0)	
Mortality, *N* (%)												
Alive	1202 (99.3)	1582 (99.5)	0.57	2015 (99.1)	2415 (99.6)	0.08	1937 (99.2)	2205 (98.8)	0.27	679 (98.0)	799 (97.4)	0.60
Dead	9 (0.7)	8 (0.5)		19 (0.9)	11 (0.5)		16 (0.8)	27 (1.2)		14 (2.0)	21 (2.6)	

Bold* p*-values represent statistically significant results

^a^Social vulnerability index quartiles; the first quartile represents the least vulnerable children and the fourth quartile represents the most vulnerable children; first quartile: 0.00 to 0.249; second quartile: 0.250 to 0.499; third quartile: 0.500 to 0.749; fourth quartile: 0.750 to 1.00

^b^When a variable with more than two groups was statistically significant, comparisons of binomial proportions were completed to understand which category was statistically significant

### Race/ethnicity

**Fig. 1:**
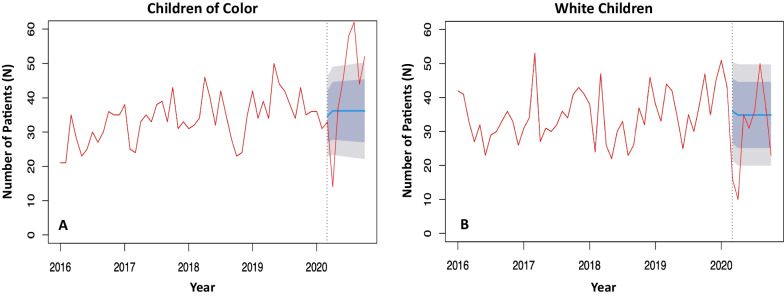
Interrupted time series analysis evaluating intentional injuries experienced by **a** children of color, and **b** White children. The red line shows the observed, monthly total of children that were intentionally injured over time. The black dashed line represents the declaration of state of emergency (March 13, 2020) in the USA regarding the COVID-19 pandemic. The blue line is the expected monthly total number of intentionally injured children over time if there were no interruption, which in this case was initiation of stay-at-home orders. The dark and light gray represent the 80% and 95% confidence interval, respectively, associated with the expected total. Comparison of density distribution by Kolmogorov–Smirnov testing and comparison of linear trends with slope analysis demonstrate a significant increase in intentional injuries for children of color during the COVID cohort compared to the Control cohort that occurred at the start of the COVID-19 pandemic (*p* = 0.007). There was no significant different in intentional injuries for White children (*p* = 0.14)

After SHO implementation, children of color experienced an increase in intentional injuries (COVID 16.4% vs. Control 13.7%, *p* = 0.03), while White children experienced no significant difference. Children of color did not experience any change in unintentional injuries between time periods; however, unintentional injuries among White children increased (COVID 95.5% vs. Control 93.7%, *p* < 0.001). An ITSA of intentionally injured children of color showed that there was a significant increase (*p* = 0.007) after SHO implementation in the observed injuries, as compared to those estimated based on monthly volume from years prior (Fig. 1a). In contrast, there was no significant increase among White children (Fig. 1b).

For type of injury, children of color experienced a decrease in blunt injuries (COVID 71.1% vs. Control 74.7%, *p* = 0.02) across time periods, while White children had an increase in burn injuries (COVID 6.2% vs. Control 4.7%, *p* = 0.002) after SHO implementation. Penetrating injuries increased in both populations; however, the relative increase in children of color was three times that in White children (children of color COVID 16.2% vs. Control 10.5%, *p* < 0.001, [5.7% increase]; White children COVID 8.9% vs. Control 7.0%, *p* = 0.002, [1.9% increase]).

There were many variations noted for children of color and White children regarding mechanism of injury between cohorts after SHO implementation. While firearm injuries among children of color almost doubled (COVID 9.0% vs. Control 5.2%, *p* < 0.001), there was no significant change in firearm injuries among White children before and after SHO implementation. Both populations were less likely to experience being struck by/against (COVID 5.7% vs. Control 9.0%, *p* < 0.001 for children of color; COVID 7.6% vs. Control 9.0%, *p* = 0.02 for White children). There were no significant differences for both children of color and White children between the COVID and Control cohort for child abuse or being cut/pierced.

Additionally, both children of color and White children were more likely to be injured from an all-terrain vehicle (ATV) crash in the COVID cohort as compared to the Control (COVID 2.0% vs. Control 1.1%, *p* = 0.04 for children of color; COVID 9.6% vs. Control 6.2%, *p* < 0.001 for White children) or a bicycle crash (COVID 6.7% vs. Control 4.8%, *p* = 0.02 for children of color; COVID 8.8% vs. Control 5.8%, *p* < 0.001 for White children). White children were less likely to be involved in a MVC in the COVID cohort (COVID 8.2% vs. Control 10.8%, *p* < 0.001), while there were no significant difference for children of color between cohorts.

### Social vulnerability

There were no significant differences in intentional injuries between the two cohorts by SVI quartile. The least vulnerable children (i.e., in the first SVI quartile) experienced more unintentional injuries in the COVID cohort (COVID 96.0% vs. Control 94.1%, *p* = 0.02), while there were no significant differences between time periods for unintentional injury in the other SVI quartiles.

For type of injury, there were no significant differences between time cohorts among the least vulnerable children in the first SVI quartile. There was an increase in burn injuries after implementation of SHOs for the third (COVID 7.8% vs. Control 5.9%, *p* = 0.02) SVI quartile. Penetrating injuries increased for the second through fourth SVI quartiles (Table 2), with the largest increase in 6.7% in the most vulnerable children (COVID 18.7% vs. Control 12.0%, *p* < 0.001).

Similar to race/ethnicity, there were significant variations for the SVI quartiles regarding mechanism of injury between cohorts. Firearm injuries increased in the second through fourth SVI quartiles (Table 2), with the greatest increase in the most vulnerable children in the fourth SVI quartile (COVID 11.1% vs. Control 6.1%, *p* = 0.001). There were no significant differences within each SVI quartile between the COVID and Control cohort for child abuse or being cut/pierced.

There were more ATV crashes for the least vulnerable populations, with increases identified in the first through third SVI quartiles, and the more pronounced increases in the first (COVID 6.5% vs. Control 4.3%, *p* = 0.01) and second (COVID 9.2% vs. Control 5.4%, *p* < 0.001) quartiles. Similarly, there was a significant increase in bicycle crashes for children living in the first (COVID 11.1% vs. Control 6.1%, *p* < 0.001) and second (COVID 8.5% vs. Control 5.6%, *p* < 0.001) SVI quartiles. MVCs decreased during the COVID cohort compared to the Control cohort for the first (COVID 6.6% vs. Control 9.3%, *p* = 0.009) and second (COVID 9.2% vs. Control 11.4%, *p* = 0.02) SVI quartiles, but there was no change in MVCs for the more vulnerable children (i.e., third and fourth quartiles).

### Injury Severity Score (ISS) and mortality

There were no significant differences between the COVID and Control cohorts for both children of color and White children regarding ISS and mortality. There were also no significant differences between the COVID and Control cohorts for all SVI quartiles for both ISS and mortality.

## Discussion

This multi-institutional study evaluated differences in pediatric traumatic injury patterns before and after implementation of SHOs between children of color and White children and across varying levels of social vulnerability based upon children’s home residence. As hypothesized, we found that children of color in the COVID cohort experienced an increase in violent injuries compared to the Control cohort, while White children did not. Similarly, children living in more vulnerable neighborhoods had an increase in violent injuries, while children living in less vulnerable neighborhoods did not. Contrary to our hypothesis, increases in non-violent mechanisms of injury, such as ATV crashes and bicycle crashes, affected all children, not just White children.

Before elaborating on these relationships, the use of race/ethnicity in this work deserves attention. As described by Trinadad and Kotagal, “race should be understood as a social construct” (Trinidad and Kotagal 2022). The findings of this study are not to suggest that race and ethnicity in and of themselves put a child at risk for injury, but that other possibly unmeasured social determinants of health are likely responsible for the variations in injury patterns. While beyond the scope of this study, we are in agreement with Trinadad and Kotagal in that “the intersection of race, racism, and socioeconomic status highlights the need to consider…how multiple social determinants of health are often interconnected, jointly impacting a child’s health.” Indeed, in one study, controlling for insurance status and Area Deprivation Index (another neighborhood-level marker of SES) led to a 56% reduction in the odds of interpersonal violence among Black children as compared to White children (Trinidad et al. 2022b).

Few studies have evaluated violent injuries and the differential impact on different races/ethnicities after the implementation of SHOs. Existing studies have largely focused on firearm injuries rather than all violent mechanisms and had mixed results. Martin et al. showed that children of color, particularly Black children, had higher increases in exposure to firearm incidents after SHO initiation compared to White children (Martin et al. 2022). Another study found that Black children were disproportionately injured by firearms after the start of SHOs, compared to before, while White and Latinx children were not (Afif et al. 2022). These findings are similar to that of Peña & Jena, who identified that areas with populations > 50% that were Black or Hispanic had more pediatric gun-related deaths than areas with populations < 50% that were Black or Hispanic (Peña and Jena 2022). However, other studies found no variation by race in pediatric firearm injuries before and after SHOs (Collings et al. 2022; Gastineau et al. 2021). The differences in findings may be explained by variation in racial/ethnic categories. Additionally, Gastineau et al. performed a national database study that utilized Pediatric Health Information System (PHIS), which may not have elucidated the nuances that are clear in our study using registry data specific to the trauma population.

Regarding SES as measured by neighborhood vulnerability, we found that children living in neighborhoods of higher vulnerability had an increase in firearm injuries compared to children living in lower-vulnerability neighborhoods after the start of SHOs. Peña and Jena also found that children living in low-income neighborhoods experienced more gun-related deaths compared to high-income neighborhoods (Peña and Jena 2022). In contrast, another study that measured SES before and after the start of SHOs through the Child Opportunity Index (COI, which measures neighborhood-level characteristics through 29 indicators through three domains of education, health and environment, and social and economic factors) found no differences in COI between proportions of children living in areas of varying opportunity that experienced firearm-related encounters (Gastineau et al. 2021; Noelke et al. 2020).

The drastic rise in firearm injuries in children of color and children living in more vulnerable neighborhoods is likely multifactorial and related to societal issues rather than the children themselves. Children living in these areas are frequently exposed to community violence and the results of structural and socioeconomic disadvantage, including low-quality education, poor housing opportunities, and poverty (Martin et al. 2022; Gastineau et al. 2021; Centers for Disease Control and Prevention 2020). The existence of these neighborhood-level disadvantages and social deprivation is due to a complex history rooted in systemic racism, harmful legislative policies, housing segregation, policing, and more (Trinidad et al. 2022b; Race 2019; Sharkey 2013). Additionally, due to school closures during the pandemic, support services, and protective factors such as mental health services, peer support, meal provision, and stable internet access were no longer accessible, which may have further exacerbated risk factors for violence (Hoffman and Miller 2020). Other features of the COVID-19 pandemic, such as the rise in firearm purchases, increased access to firearms due to a change in storage practices, and variations in state gun laws, may have also contributed (Donnelly et al. 2022; Federal Bureau of Investigation 2022; Sokol et al. 2021).

Notably, our study identified that intentional injuries increased for children of color during the COVID time period when compared to the Control time period, while unintentional injuries increased for White children and for children living in neighborhoods of lowest vulnerability. Similarly, penetrating injuries increased for children of color and children living in more vulnerable neighborhoods. Additionally, child abuse did not significantly differ before and after SHO implementation when stratified by children of color, White children, or children living in neighborhoods of varying vulnerability. These findings are consistent with prior work showing either no difference or a decrease in child abuse within the first six months of the COVID-19 pandemic (Bessoff et al. 2021; Sanford et al. 2021; Haddadin et al. 2021). This lack of increase may have been due to underreporting since children were isolated at home and professionals, such as teachers and healthcare providers, were therefore prevented from noticing any concerns (Shi et al. 2021). The change in burn injuries seen in this study, particularly the increase in White children but not children of color or the more vulnerable, could be due to the latter groups being less likely to present to a hospital due to mistrust in the hospital system, less access to care, financial strain, and the disproportionate impact of the COVID-19 pandemic rather than a true lack of injury burden (Sanford et al. 2021; Abuelgasim et al. 2020; Thakur et al. 2020).

Many studies have shown either a decrease or no significant difference in pediatric MVC injuries and an increase in ATV and bicycle crashes after the start of SHOs (Flynn-O’Brien et al. 2022a; Bessoff et al. 2021; Sanford et al. 2021; Yeates et al. 2022; Haddadin et al. 2021). Our study is the first to show that while MVCs decreased for White children and children living in areas of lower vulnerability after SHO initiation, they did not decrease for children of color or children living in areas of high vulnerability. Overall, the injuries that primarily and preferentially affected White children and children in areas of lower vulnerability were ATV and bicycle crashes. Hence, while overall injury burden was felt by all children, the types of injuries sustained were still disparately distributed.

The onset of the COVID-19 pandemic and SHOs caused people, including children, to remain at home more, leading to a drastic and sustained decline in road travel and motor vehicle use (Gross 2021). Since children no longer had school or other extracurricular activities to occupy their time and the outdoors was considered to be safer than indoors, the rise in ATV and bicycle crashes was perhaps expected. The warm summer months may have further exacerbated these increases. The higher occurrence of these injuries in White children and children in neighborhoods of lower vulnerability is reflective of the historic trend of increased utilization of ATVs and bicycles in this population (Brown et al. 2002; Jennissen et al. 2022). Targeted education about safety with utilization of ATVs and bicycles is critical for prevention of injuries. The increase in injuries may have been due to closures of a broad array of public services and healthcare environments, education and training courses regarding safe use (i.e., wearing a helmet or age recommendations for riding).

Due to the retrospective nature of this study, it may be limited by misclassification bias, selection bias, recording errors, and unknown/missing data. Additionally, since our study included children that met NTDB criteria, children that were not transferred and/or discharged from the Emergency Department or that died prior to arrival were excluded. Therefore, children with minor injuries may not have been captured. However, this excludes the potential for erroneous data that can exist with utilization of institutional ICD-10 codes for all emergency department encounters. Regarding SVI, there is risk of misclassification bias since it is a metric that uses granular census tract data rather than zip codes. Furthermore, since the United States Census Bureau releases new SVI data every two years, SVI could have theoretically changed in the interim. However, this would have affected both the Control and COVID cohorts. Lastly, since children with an unknown race/ethnicity were excluded from the analysis, there is a risk of over or underestimating the true differences.

Additionally, the definition of all firearm injuries and intentional injuries as ‘violent’ injuries must take into account discrepancies in terminology and etiology of injury. Though there are some firearm injuries that may not be considered violent due to a lack of assaultive intent (i.e., a young child accidentally injuring themselves with an unsafely-stored firearm), we reasoned that these instances still constitute a violent injury due to the nature of the injury. Similarly, though some firearm injuries may be labeled as unintentional, this may not truly reflect the nuances of the circumstances (i.e., a child accidentally injured by a stray bullet going through a home could potentially be classified as an unintentional injury, but the firearm was intentionally fired). These reasons are why we ultimately defined ‘violent’ injuries as including both firearm and intentional injuries. However, we recognize there are limitations to the varying terminologies.

## Conclusion

In conclusion, injured children of color and children living in neighborhoods of higher vulnerability experienced a significant increase in violent injuries during the COVID-19 pandemic in the USA, while White children and children from neighborhoods of lower vulnerability did not. Addressing these disparities is essential to mitigate the impact of future pandemic effects on children. Additionally, differences in non-violent mechanisms, such as bicycle and ATV crashes, that occurred across race/ethnicity and neighborhood vulnerability were likely reflective of changes in the social environment and extracurricular activities of children. Education regarding these mechanisms of injury is important for all populations. Identifying specific trends of pediatric trauma and those affected is an initial step toward targeted intervention strategies.

### Supplementary Information

**Additional file 1.** Analysis of variance testing for effect of the COVID-19 pandemic on race/ethnicity and social vulnerability with injury patterns.

## Data Availability

Available in tables or as supplementary material; there are no data repositories.

## Abbreviations

SHOs: Stay-at-Home orders
SES: Socioeconomic status
SVI: Social Vulnerability Index
NTDB: National Trauma Data Bank
ICD-10: International Classification of Diseases, 10th revision
MVC: Motor vehicle crash
ISS: Injury Severity Score
ITSA: Interrupted time series analysis
ATV: All-terrain vehicle
COI: Child Opportunity Index
