# Supplementary material for: Relationship between the COVID-19 pandemic and structural inequalities within the pediatric trauma population
Source: Inj Epidemiol. 2023 Nov 28;10(Suppl 1):62. doi: 10.1186/s40621-023-00475-0 (PMC10683076; doi:10.1186/s40621-023-00475-0)
Supplement: Supplementary file 1 — Additional file 1. Analysis of variance testing for effect of the COVID-19 pandemic on race/ethnicity and social vulnerability with injury patterns. [file 40621_2023_475_MOESM1_ESM.docx]

| **Additional File 1. Analysis of variance testing for effect of the COVID-19 pandemic on race/ethnicity and social vulnerability with injury patterns** | | |
| --- | --- | --- |
|  | **Chi-squared Likelihood Ratio** | **P-value** |
| **Race/Ethnicity** |  |  |
| Intent of Injury | 20.3 | **<0.001** |
| Type of Injury | 12.2 | 0.06 |
| Mechanism of Injury | 56.9 | **<0.001** |
| **Social Vulnerability** |  |  |
| Intent of Injury | 8.6 | 0.20 |
| Type of Injury | 11.3 | 0.25 |
| Mechanism of Injury | 50.6 | **0.01** |
